# Acceptability of fully guided virtual implant planning software among dental undergraduate students

**DOI:** 10.1186/s12903-023-03064-1

**Published:** 2023-05-29

**Authors:** Shishir Ram Shetty, Colin Murray, Sausan Al Kawas, Sara Jaser, Wael Talaat, Medhini Madi, Vinayak Kamath, Nisha Manila, Raghavendra Shetty, Vidya Ajila

**Affiliations:** 1 College of Dental Medicine, University of Sharjah, Sharjah, United Arab Emirates; 2 Department of Oral and Cranio-facial health Sciences, University of Sharjah, Sharjah, United Arab Emirates; 3 A. B. Shetty Memorial Institute of Dental Sciences, Nitte (Deemed to be University), Mangalore, India; 4 Manipal College of Dental Sciences, Manipal, Manipal Academy of Higher Education, Manipal, India; 5 Goa Dental College and Hospital, Bambolim, Goa India; 6 School of Dentistry, Louisiana State University Health Science Centre, New Orleans, LA United States of America; 7 College of Dentistry, Ajman University, Ajman, United Arab Emirates; 8 Datta Meghe Institute of Higher Education and Research (Declared as Deemed-to-be University), Maharashtra, India; 9 Nitte (Deemed to be University), Mangalore, India

**Keywords:** 3D files, Virtual implant planning software, Dental education

## Abstract

**Background:**

Fully guided implant surgery as a technique is gaining popularity. It has been observed that use of surgical guides improves precision and predictability for dental implant placement. However, like any other newer technology, the acceptance of fully guided dental implant technology among users is based upon its perceived usability. This study aimed at evaluating the perception about using Virtual Implant Planning Software (VIPS) among undergraduate dental students at the university of Sharjah.

**Methods:**

Ninety-Six dental surgery students from the University of Sharjah were included in the study. One week after the Virtual Implant Planning Software (Planmeca Romexis version 6.2 procedure, students were asked to complete a Combined technology acceptance model and the theory of planned behaviour (C-TAM TPB) questionnaire. Sixty-six students responded to the questionnaire.

**Results:**

Cronbach’s alpha surpassed 0.7 for perceived usefulness, perceived ease of use, perceived behavioral control, and subjective norm. Attitude and behavioural intention reported Cronbach’s alpha values less than 0.7. Spearman’s correlation coefficient was significant for all the constructs. Perceived ease of use explained 49%, 33%, and 42% of the variance of perceived usefulness (R^2^ = 0.49), attitude (R^2^ = 0.33), and perceived behavioral control (R^2^ = 0.42) respectively. Perceived usefulness explained 25%, 18%, and 23% of the variance of attitude (R^2^ = 0.25), behavioral intention (R^2^ = 0.18), and perceived behavioral control (R^2^ = 0.23) respectively. Attitude accounted for 25%, 33%, and 29% of the variance of behavioral intention (R^2^ = 0.25), perceived behavioral control (R = 0.33), and subjective norm (R = 0.29) respectively.

**Conclusion:**

The fully guided VIPS was acceptable by dental students specifically because of its usability. This makes VIPS a very effective tool for teaching implantology for dental students. VIPS also allows students to perform multiple repetitions of the implant planning procedure which enhances understanding and content retention.

## Background

The use of immersive and non-immersive 3D educational files has been receiving immense attention in medical education over recent years [1]. Intraoral scan (IS) and cone beam tomography (CBCT) are 3D files that have got potential to be useful 3D educational material in dentistry [2]. In the past, 3D files were considered out-of-reach educational equipment since they required advanced technological and human resources that were not available [2]. Nonetheless, in the medical field presently, sophisticated imaging devices such as an intraoral scanner (IS) and Cone Beam Computed Tomography (CBCT) have been developed particularly in dentistry for various clinical applications [2]. These devices are projected to open various applications via the use of digitalized 3D files [2, 3]. Additionally, these devices have the potential to improve the ability of medical and dental students of learning subjects like anatomy [2].

The development of cone beam tomography (CBCT) and the invention of interactive software to permit virtual planning to guide surgery precisely towards a specific target has significantly improved oral surgery [4]. Virtual implant planning software (VIPS) permits prosthetically driven methods leading to better prosthesis design, esthetics-optimized occlusion, and loading [4].

Several key factors determine the success of implant-based rehabilitations. These factors include nature of oral tissues (both hard and soft), systematic condition of the patient and implant maintenance (oral hygiene and bacterial microleakage) in the long run [5–10]. Implant selection (micro and macro implant factors, neck design), implant positioning (tilt), and several other implant related factors also influence the success of implant based oral rehabilitations [11, 12].

VIPS enables us to perform guided oral rehabilitation with advantages such as minimizing surgical trauma and complications. However, VIPS can be susceptible to designing errors which can affect the rehabilitation procedure [5, 6].

Intraoral scan (IS) can capture the form, shape and structure of oral soft tissues, and the teeth [4]. The blend of cone beam tomography (CBCT) and Intraoral scan (IS) images, by mutual superposing and use of virtual implant planning software, presents an absolute 3D depiction of hard and soft tissues. Additionally, new planning software permits the development of a digital wax-up of the future prosthetic plan, which can be visualized and modified if deemed fit [4]. Based on such a complete set of information the design and fabrication of computer surgical templates can progress with sufficient accuracy which can result in more precise implant positioning than obtained in previous techniques [4].

Institutions of higher learning and healthcare spend a lot of resources on novel technologies [13]. The adoption of the invention is a crucial investment choice [14]. In the past, technology acceptance has been studied from different theoretical viewpoints [14]. The studies suggested that acceptance behavior is affected by social and situational influences, user beliefs, attitudes, and leadership interventions [14].

Recently published research articles have stated that virtual implant planning software (VIPS) can improve implant surgical training in dental students [13, 15]. However, it is very important to understand how the students perceive the use of newer technologies like VIPS worldwide since they will be future users of this software. Researchers have used a combined technology acceptance model and the theory of planned behavior (C-TAM TPB) to obtain student feedback while introducing newer technology in education [14].

To the best of our knowledge, there is only one study conducted in Germany which explores this area of research [13]. Furthermore, there is no evidence in any published literature regarding the acceptability of fully guided implant planning software among dental undergraduate students. Therefore, this study aimed at evaluating the perception about using Virtual Implant Planning Software (VIPS) among undergraduate dental students at the university of Sharjah.

## Methods

**Fig. 1:**
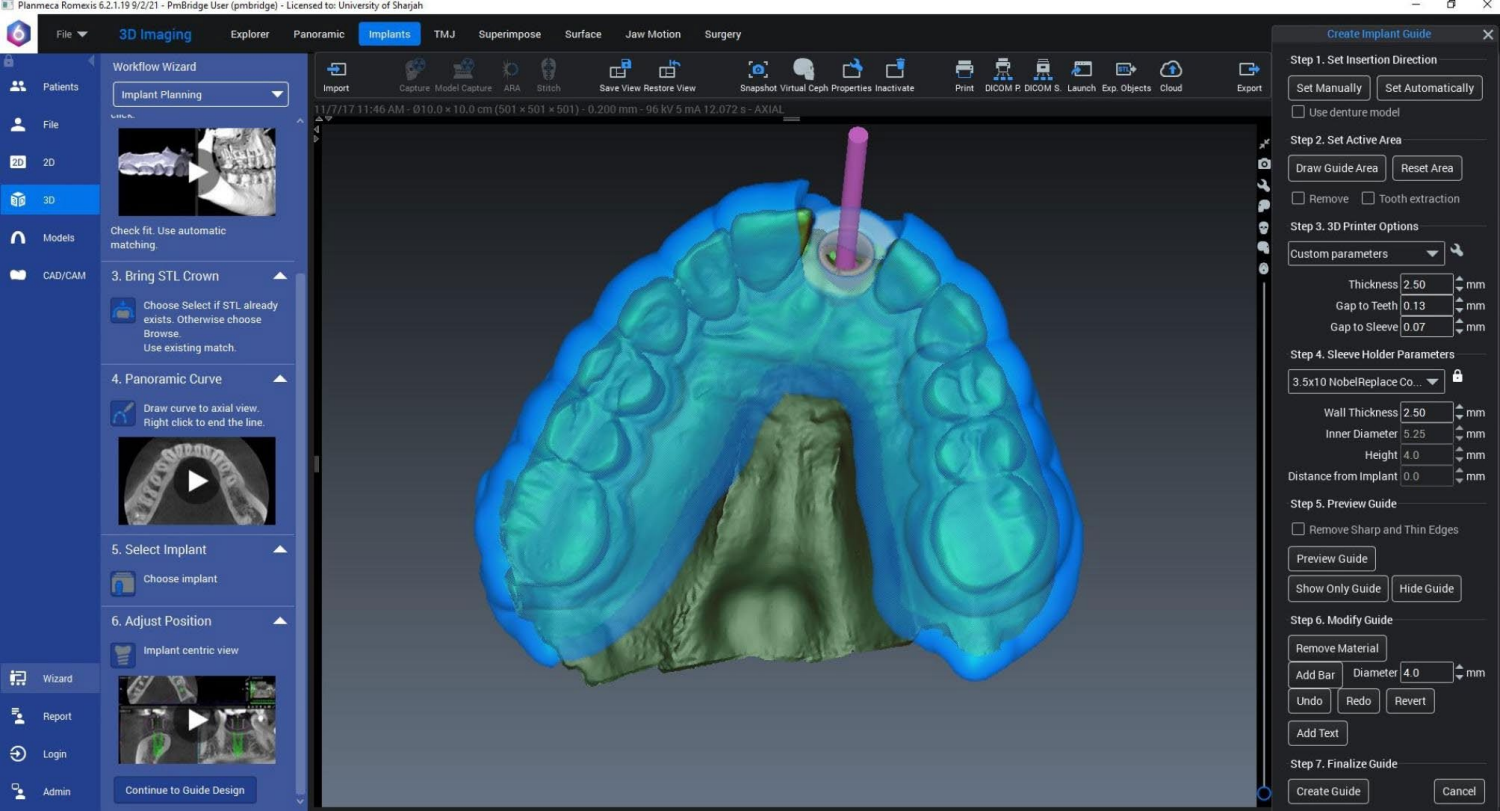
Surgical guide prepared by the students using the ROMEXIS® virtual implant planning software

Ninety-Six fifth-year Bachelor of Dental surgery (BDS) students from the College of Dental Medicine, University of Sharjah were included in the study. Ethical Approval was obtained from the University of Sharjah, Research Ethics Committee (REC-22-04-24). The implant radiology theory was delivered to the students’ lecture as a part of the Dental Health Science (DHS) 5 course. The students were provided with Virtual implant planning (VIP) training videos one week before the hands-on training session. A faculty with 10 years of teaching experience in oral radiology assisted the students to perform one fully guided VIP procedure **(**Fig. 1**)**.

The student then repeated the procedure without any assistance from the faculty. The faculty provided feedback to the student about the procedure.

One week after the VIP procedure, students were asked to complete a Combined technology acceptance model and the theory of planned behaviour (C-TAM TPB) questionnaires [14]. The questionnaires had 28 measurement items. The first 6 sets of measurement items were categorized under the construct of Perceived Usefulness, the next 6 were Perceived Ease of Use, the next 5 Perceived Behavioral Control, the next 6 Subjective Norm, followed by 3 and 2 for Attitude, and Behavioral Intention constructs respectively. The items were measured on a six-point Likert scale requiring agreement or disagreement [16]. The scale extended from 1 (“I strongly disagree”) to 6 (I strongly agree) [16].

### Statistical analysis

Percentiles and Median values were computed for the outcome of the survey. Mean and standard deviations were also calculated for statistical data. Cronbach’s alpha evaluation was undertaken to determine if Likert scale questionnaires were internally consistent [17]. Cronbach’s alpha coefficient was evaluated for all the constructs. Cronbach’s alpha values between 0.7 and 0.95 are within the permitted internal consistency range [17]. Spearmen’s correlation coefficients were computed to assess the strength and direction of association between ranked variables. Squared multiple correlations were evaluated to determine the proportion of variance in the dependent variable that can be accounted for by the independent variable. All computations were done using Microsoft Excel Spreadsheet.

## Results

Sixty-six questionnaires were completed and used for data evaluation. The outcomes for different items are in Table 1.

**Table 1:** Measurement Items of the questionnaire used in the study

	Measurement Items.	N	Mean	SD	Min	Max	Percentiles		
							Q1	Median	Q3
Perceived Usefulness	The implant planning software enables me to accomplish tasks more quickly.	66	5.70	0.94	1	6	6	6	6
	The implant planning software has improved my quality of work.	66	5.36	1.26	1	6	5	6	6
	The implant planning software makes it easier to do my job.	66	5.61	0.99	1	6	6	6	6
	The implant planning software has improved my productivity.	66	5.58	0.99	1	6	5.75	6	6
	The implant planning software gives me greater control over my job.	66	5.58	0.99	1	6	5.75	6	6
	The implant planning software enhances my effectiveness on the job.	66	5.58	0.99	1	6	5.75	6	6
Perceived Ease of Use	My interaction with the implant planning software has been clear and understandable.	66	5.61	0.96	1	6	5.75	6	6
	Overall, the implant planning software is easy to use.	66	5.55	0.93	1	6	5	6	6
	Learning to operate the implant planning software was easy for me.	66	5.55	0.86	2	6	5	6	6
	I rarely become confused when I use implant planning software.	66	5.36	1.02	2	6	5	6	6
	I rarely make errors when using implant planning software.	66	5.24	1.08	3	6	4.75	6	6
	I am rarely frustrated when using the implant planning software	66	5.30	1.20	1	6	5	6	6
Perceived Behavioral Control	I am able to confidently use the implant planning software.	66	5.33	1.04	3	6	5	6	6
	I have the knowledge to use implant planning software.	66	5.55	0.83	3	6	5	6	6
	I have the resources to use the implant planning software.	66	5.15	1.32	2	6	4.75	6	6
	I have the ability to use implant planning software.	66	5.45	0.93	3	6	5	6	6
	I have control over using the implant planning software.	66	5.36	1.02	2	6	5	6	6
Subjective Norm	People who influence my behavior think I should use implant planning software.	66	5.39	0.99	2	6	5	6	6
	People who are important to me think I should use implant planning software.	66	5.45	0.90	2	6	5	6	6
	My immediate supervisor thinks I should use the implant planning system.	66	5.30	1.20	2	6	5	6	6
	My close friends think I should use the implant planning system.	66	5.24	1.29	1	6	5	6	6
	My peers think I should use the implant planning system.	66	5.36	1.08	2	6	5	6	6
	People whose opinions I value prefer that I use implant planning software in my work.	66	5.48	1.00	2	6	5	6	6
Attitude	Using the implant planning software is a good idea.	66	4.82	0.46	3	5	5	5	5
	Using the implant planning software is unpleasant.	66	3.73	2.41	1	6	1	5	6
	Using the implant planning software is beneficial to patient care	66	5.82	0.46	4	6	6	6	6
Behavioral Intention	I intend to continue using implant planning software to perform my job.	66	5.73	0.67	3	6	6	6	6
	I intend to frequently use implant planning software to perform my job.	66	5.58	0.99	1	6	5.75	6	6

Cronbach’s alpha surpassed 0.7 for Perceived Usefulness, Perceived Ease of Use, Perceived Behavioural Control, and Subjective Norm. Attitude and Behavioural Intention reported Cronbach’s alpha values less than 0.7 **(**Table 2**).**

**Table 2:** Consistency of the Subscales in relation to responses

Subscales	N	Mean	SD	Min	Max	Percentiles			Cronbach’s Alpha
						Q1	Median	Q3	
Perceived Usefulness	66	33.39	5.78	6	36	31.75	36	36	0.97
Perceived Ease of Use	66	32.61	5.13	12	36	30	36	36	0.92
Perceived Behavioral Control	66	26.85	4.35	15	30	25	30	30	0.89
Subjective Norm	66	32.24	5.94	12	36	30	36	36	0.96
Attitude	66	14.36	2.68	10	17	12	14	17	0.67
Behavioral Intention	66	11.30	1.44	7	12	11.75	12	12	0.61

**Fig. 2:**
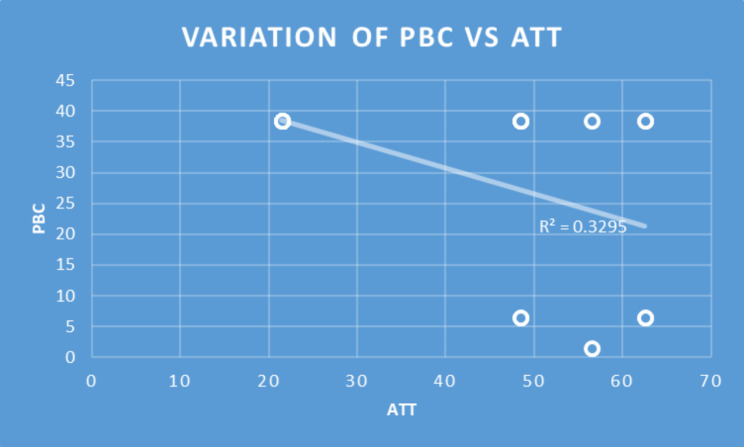
Correlation of Perceived Behavioral Control (PBC) and Attitude (ATT)

**Fig. 3:**
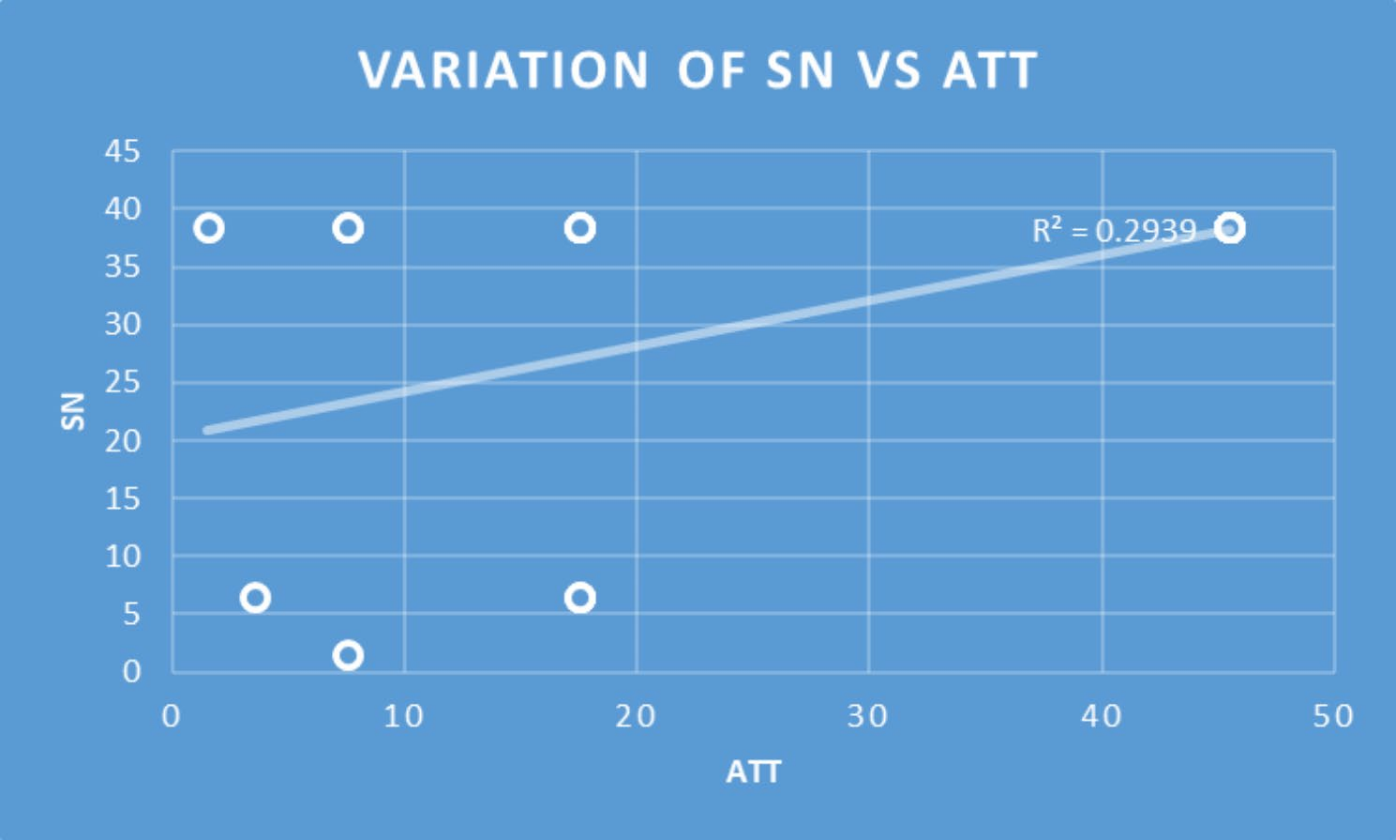
Correlation of Subjective Norm (SN) and Attitude (ATT)

**Fig. 4:**
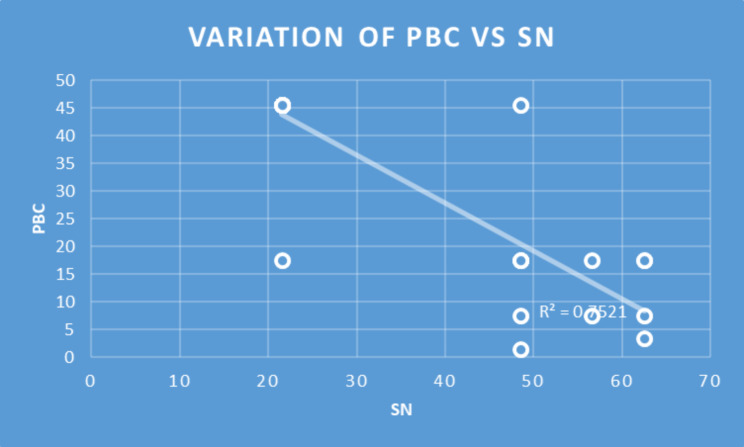
Correlation of Perceived Behavioral Control and Subjective Norm

Spearman’s correlations were significant for all the constructs (Table 3). Only selected pairs of independent and dependent variables deemed relevant were included in Table 3. Perceived Ease of Use explained 49%, 33%, and 42% of the variance of Perceived Usefulness (R^2^ = 0.49), Attitude (R^2^ = 0.33), and Perceived Behavioral Control (R^2^ = 0.42) respectively. Perceived Usefulness explained 25%, 18%, and 23% of the variance of Attitude (R^2^ = 0.25), Behavioral Intention (R^2^ = 0.18), and Perceived Behavioral Control (R^2^ = 0.23) respectively. Attitude accounted for 25%, 33%, and 29% of the variance of Behavioral Intention (R^2^ = 0.25), Perceived Behavioral Control (R = 0.33), and Subjective Norm (R = 0.29) respectively **(**Figs. 2, 3 and 4**)**.

**Table 3:** Spearman’s Correlation Test

Independent Variables	Dependent Variables	Spearman’s Correlation (R)	p-values	Squared Multiple Correlation(R^2^)
Perceived Ease of Use	Perceived Usefulness	0.70	< 0.001*	0.49
Perceived Ease of Use	Attitude	0.57	< 0.001*	0.33
Perceived Ease of Use	Perceived Behavioral Control	0.65	< 0.001*	0.42
Perceived Usefulness	Attitude	0.50	< 0.001*	0.25
Perceived Usefulness	Behavioral Intention	0.42	< 0.001*	0.18
Perceived Usefulness	Perceived Behavioral Control	0.48	< 0.001*	0.23
Attitude	Behavioral Intention	0.50	< 0.001*	0.25
Attitude	Perceived Behavioral Control	-0.57	< 0.001*	0.33
Attitude	Subjective Norm	0.54	< 0.001*	0.29

*p < 0.05 Statistically Significant, p > 0.05 Non-Significant, NS

## Discussion

Acceptance of new technology is a challenge in the health care sector [18]. Previous studies, on the impact of new technologies in healthcare, reveal that the fit between the technology and the clinical work significantly affects how the target users will reject or accept and incorporate it into their daily practice or work around it [18].

Dental implants have progressively become a crucial part of reconstructive dentistry [15]. In many applications implants provide a viable choice for replacement of missing teeth [15]. Dental students must therefore equip themselves with relevant knowledge about dental implantology technology and be informed of situations where implants may be appropriate during the different phases of treatment planning and execution [19].

Fully guided virtual implant planning software permits users to simulate the location of an implant in 2D and 3D models, identify the inferior alveolar dental canal, trace panoramic views, and calculate bone density [15, 20]. A viable example of a fully guided implant planning software that has been applied for teaching and learning at the undergraduate level combines an immersive head-mounted display, a small hand-tracking device, and a smartphone. The devices are connected to a laptop to provide the user interface. The user’s dominant hand is used to manipulate the 3D dental models while the remaining hand holds the controller (smartphone) to ensure accurate positioning and inclination during implant input [20].

**Fig. 5:**
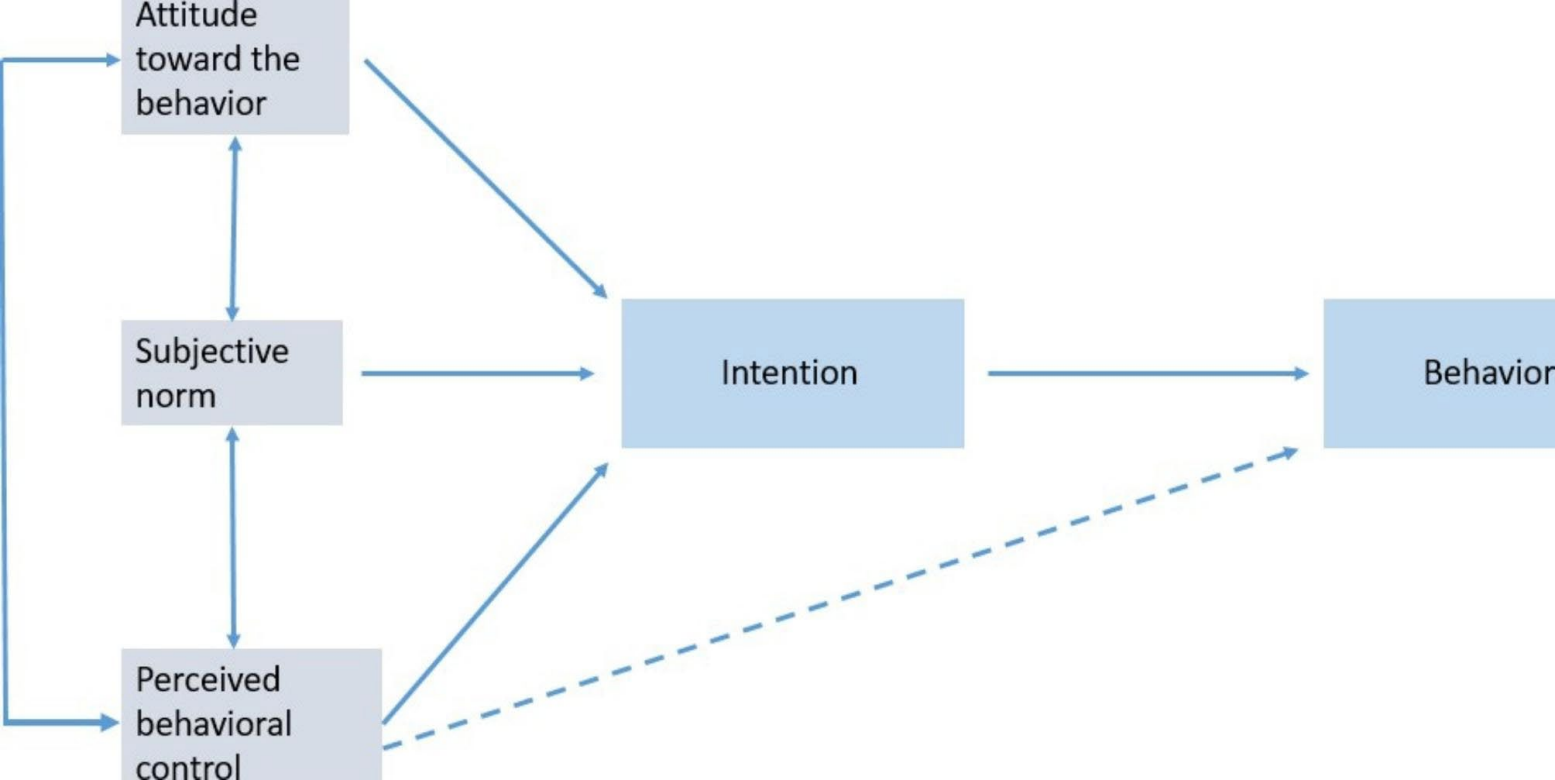
Schematic representation of Arjen’s Theory of Planned Behavior [27]

The adoption of novel technologies is expected to rise in dentistry and with such advances comes the problem of how best to implement them in dental education. The greatest challenge still lies with the capacity to recognize, envisage, and control the acceptance of a novel technology because this will affect its implementation [21]. Several technology acceptance models have been developed [22, 23]. A classic example of a technology acceptance model that is of particular interest in the health sector is the theory of planned behavior (TPB) [24]. It is a modified theory of reasoned action that extends beyond the theory of reasoned action to incorporate the idea of perceived behavioral control [25, 26]. It takes into consideration perceived and actual management of the behavior under consideration [26]. Perceived behavioral control affects attitude and intention and can also directly influence behavior [26]. Attitudes towards a certain behavior are a revelation of an individual’s assessment of performing a given behavior. Subjective norm shows an individual’s perception of societal expectations to assume a certain behavior [26]. Perceived behavioral control shows the ease or complexity with which performing the behavior is likely to be. It mirrors both internal and external factors such as the availability of time and resources. Arjen’s Theory of Planned Behavior is commonly used because it performs well across behavioral categories regarding explaining intentions [27] (Fig. 5).

Technology Acceptance Model (TAM) also originates from the theory of reasoned action [28, 29]. TAM has two principal factors affecting an individual’s intention to adopt a novel technology: Perceived Ease of Use and Perceived Usefulness [28, 29]. For instance, a person who believes in the perception that digital games as too challenging to play would be unlikely to use this technology while one who perceives digital games as interesting and easy to learn would be more likely to learn how to use the game [25]. Integrating TAM and TPB models provides greater flexibility especially when investigating factors influencing intention [24]. Due to the technical combinations of various constructs used in this study, the combined model of technology acceptance model and theory of planned behavior (C-TAM-TPB) was adopted.

Internal consistency of measurements of various constructs compared well with similar trials in the healthcare sector. Cronbach’s alpha surpassed 0.7 for Perceived Usefulness, Perceived Ease of Use, Perceived Behavioral Control, and Subjective Norm representing an acceptable internal consistency [9]. However, Attitude and Behavioural Intention reported Cronbach’s alpha values less than 0.7. The low values of Cronbach’s alpha could be due to the low number of questions in the constructs. For instance, Behavioral Intention only had 2 sets of questions (the least) followed by Attitude which had 3 sets of questions. This did not compare fairly with other constructs such as perceived ease of use which had up to 6 sets of questions. The results of our study were consistent with findings of the study by Nkenke et al. using the basic implant planning software [13]. In this study, statistical data such as age, gender or year of study had limited relevance and hence were not included in the statistical evaluation. Spearman’s correlations were significant for all the constructs, especially for useability (Perceived ease of use). Similar findings were reported in the study by Nkenke et al. among German dental students. It can therefore be inferred that that the perceived ease of use and acceptance of VIPS among dental students is not restricted to specific geographic boundaries.

Furthermore, several undergraduate and postgraduate programs incorporate virtual learning technologies in teaching primarily because there are no adverse clinical consequences associated with [30]. Additionally, virtual technologies permit multiple repetitions without additional cost of materials which are essential for learning and the practice of new concepts [31].

The present study has some limitations. There are not many studies on the acceptability of newer dental technologies, especially VIPS. This makes it difficult to compare the findings of our study with similar research papers. Secondly, the present study was conducted on a relatively small sample size of students which makes it hard to generalize the findings of the study.

## Conclusion

Fully guided virtual implant planning software was deemed to be acceptable by dental students specifically because of its usability. Virtual technologies should be developed as part of teaching and learning within the dental curriculum as they permit multiple repetitions which enhances understanding and content retention. Future studies can be carried out evaluate the educational outcomes of VIPS among students.

## Data Availability

The corresponding author (shishirshettyomr@gamil.com) can be contacted for raw data. The data can also be accessed at **figshare** using the below link. https://figshare.com/articles/dataset/Acceptability_of_Fully_Guided_Virtual_Implant_Planning_Software_Among_Dental_Undergraduate_Students/21802374.
